# Isopropyl 3-(3,4-Dihydroxyphenyl)-2-hydroxypropanoate Alleviates Palmitic Acid-Induced Vascular Aging in HUVEC Cells through ROS/Ferroptosis Pathway

**DOI:** 10.3390/ijms25179278

**Published:** 2024-08-27

**Authors:** Xin He, Xiaohui Zheng, Weidong Xie

**Affiliations:** 1 State Key Laboratory of Chemical Oncogenomics, Shenzhen International Graduate School, Tsinghua University, Shenzhen 518055, China; hexingood56@163.com; 2 Open FIESTA Center, Shenzhen International Graduate School, Tsinghua University, Shenzhen 518055, China; 3 Shenzhen Key Laboratory of Health Science and Technology, Institute of Biopharmaceutical and Health, Tsinghua University, Shenzhen 518055, China; 4 School of Life Sciences, Northwestern University, Xi’an 710069, China

**Keywords:** palmitic acid, IDHP, aging, ROS, ferroptosis

## Abstract

Vascular aging is an important factor leading to cardiovascular diseases such as hypertension and atherosclerosis. Hyperlipidemia or fat accumulation may play an important role in vascular aging and cardiovascular disease. Isopropyl 3-(3,4-dihydroxyphenyl)-2-hydroxypropanoate (IDHP) has biological activity and can exert cardiovascular protection, which may be related to ferroptosis. However, the exact mechanism remains undefined. We hypothesized that IDHP may have a protective effect on blood vessels by regulating vascular aging caused by hyperlipidemia or vascular wall fat accumulation. The aim of this study is to investigate the protective effect and mechanism of IDHP on palmitic acid-induced human umbilical vein endothelial cells (HUVEC) based on senescence and ferroptosis. We found that IDHP could delay vascular aging, reduce the degree of ferrous ion accumulation and lipid peroxidation, and protect vascular cells from injury. These effects may be achieved by attenuating excessive reactive oxygen species (ROS) and ferroptosis signaling pathways generated in vascular endothelial cells. In short, our study identified IDHP as one of the antioxidant agents to slow down lipotoxicity-induced vascular senescence through the ROS/ferroptosis pathway. IDHP has new medicinal value and provides a new therapeutic idea for delaying vascular aging in patients with dyslipidemia.

## 1. Introduction

In modern society, the issue of aging has attracted more and more public attention. Aging is a complex biological process, and structural and functional aging can lead to the gradual aging of multiple organs. Cellular senescence, the irreversible cessation of cell division, is closely related to the aging process of an individual [1]. Effective treatment strategies can delay the aging of blood vessels [2,3]. However, the mechanisms of vascular aging and the ideal drugs remain uncertain.

Vascular remodeling, endothelial dysfunction, and loss of vascular function are important features of aging [4,5]. The gradual loss of vascular function during the aging process is an important factor leading to the high incidence of cardiovascular diseases in the elderly [6,7]. The rate of aging depends on many factors, including internal and external factors [8]. In addition to age, pathophysiological and biochemical changes such as the increase in free radicals and inflammation will also accelerate the process of cell aging [9]. Aging of blood vessels will lead to myocardial stiffness and may also lead to other diseases [10], such as heart failure, arrhythmias, and sudden cardiac death [11]. In addition, aging of blood vessels may increase the risk of stroke and lead to a decline in immunity [12].

Cardiovascular disease (CVD) is the leading cause of death worldwide. Vascular aging, that is, degenerative changes in blood vessels, is one of the important risk factors for CVD. In addition to age factors, environmental factors such as diet, smoking, high blood pressure, and other factors can also affect vascular aging. Furthermore, hyperlipidemia or fat accumulation may play a key role in mediating vascular aging [13].

Ferroptosis is involved in the metal iron non-apoptotic cell death [14]. It is a new type of programmed cell death dependent on iron concentration. Most mechanisms of ferroptosis are related to lipid peroxidation. The hallmark features of ferroptosis, which is dependent on intracellular ferrous ions, include intracellular lipid peroxidation and ferrous accumulation [15,16].

Ferroptosis has a strong negative impact on vascular aging. The increased degree of ferroptosis will lead to vascular aging. Eliminating vascular ferroptosis signal in age-related cardiovascular diseases is an important idea to resist vascular aging [17,18,19]. During ferroptosis, ROS is able to activate several transcription factors and protein kinases that promote ferroptosis [19,20]. ROS also inhibits some resistance to iron the signal molecules of death, so as to regulate the process of iron death. Mitochondria are involved in the regulation of these signaling pathways by producing ROS and other signaling molecules [18,21,22,23]. Furthermore, GPX4 is a key protein in ferroptosis, which depends on the content of GSH. Detecting the expression levels of GSH and GPX4 is crucial in detecting the degree of ferroptosis [24]. Iron abnormalities in cells can lead to lipid peroxidation and GSH depletion, leading to ferroptosis of cells.

**Figure 1:**
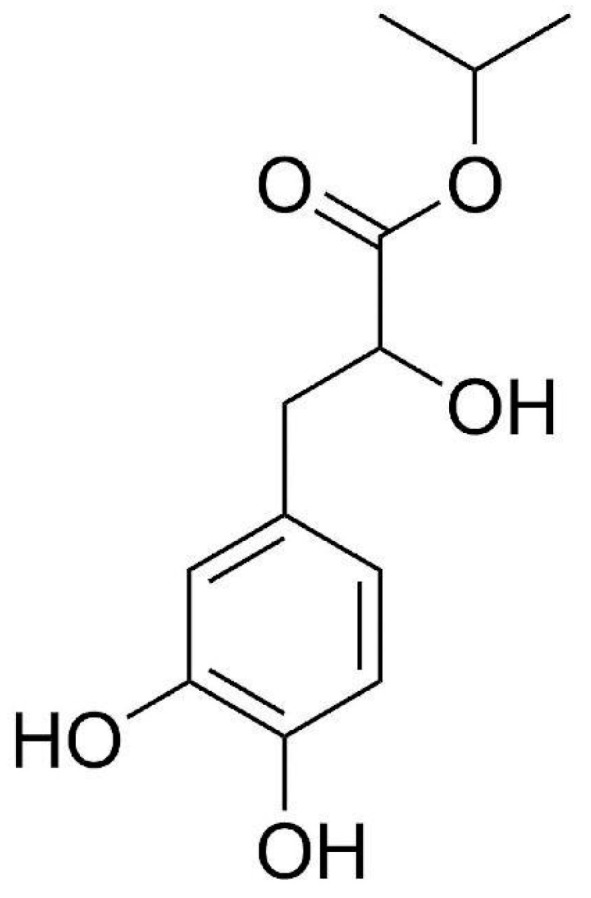
Isopropyl 3-(3,4-dihydroxyphenyl)-2-hydroxypropanoate 2D structure.

Isopropyl 3-(3,4-dihydroxyphenyl)-2-hydroxypropanoate (IDHP) is one of the main bioactive metabolites of the Chinese medicinal herb Danshen, 2D structure diagram as shown in Figure 1 [25]. IDHP can slow down oxidative damage. Recent studies have shown that IDHP has antioxidant and anti-inflammatory effects, and can also inhibit cell cycle and reduce mitochondrial dysfunction [26,27]. In our previous study, we found that PA can induce HUVEC cell aging, and IDHP has potential for the vascular aging model induced by PA. However, the specific mechanism remains unclear and requires further investigation.

In this study, we hypothesized that IDHP may have a role in delaying vascular senescence caused by hyperlipidemia and have a protective effect against cardiovascular disease. We investigated the role of IDHP in delaying PA-induced cellular senescence from SA-β-gal, ROS experiment, GSH, and the important index of iron death ferrous ion accumulation degree and Lipid ROS Lipid peroxidation levels. With these, we identified the mechanism by which IDHP regulates cellular senescence via the ROS/ferroptosis pathway.

## 2. Results

### 2.1. Experimental Results of MTT

**Figure 2:**
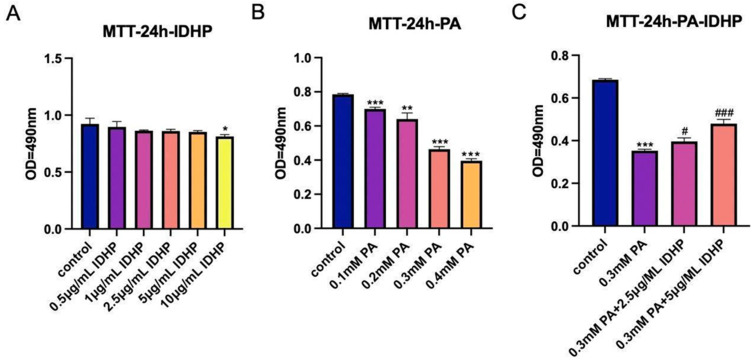
Experimental results of MTT. (**A**) MTT assay results for 24 h of IDHP administration, (**B**) MTT assay results for 24 h of PA administration, (**C**) MTT assay results for 24 h of IDHP and PA administration. Data are expressed as the means ± SD. * represents *p* < 0.05, compared to control group; ** represents *p* < 0.01 to control group; *** represents *p* < 0.001 to control group; # represents *p* < 0.05, compared to PA group; ### represents *p* < 0.001 compared to PA group, n = 5.

We found out the dose of non-toxic IDHP by administering IDHP for 24 h. We found that IDHP had a significant effect on HUVEC cells at 10 μg/ML. For our subsequent experimental concentrations, IDHP of 2.5 μg/ML and 5 μg/ML was selected as the experimental drug concentration, as shown in Figure 2A. Then, we used PA to establish the aging model. In order to significantly present the aging model, we selected a PA concentration that was approximately 40% cell lethality. This concentration was 0.3 mM, as shown in Figure 2B. Next, we used PA to induce senescence and then administered IHDP to test the effect of IDHP on cell viability. We found that IDHP can alleviate the cell senescence caused by PA in a dose-dependent manner. IDHP has a certain effect on relieving cell senescence, as shown in Figure 2C.

### 2.2. IDHP Delays PA-Induced HUVEC Cell Senescence

**Figure 3:**
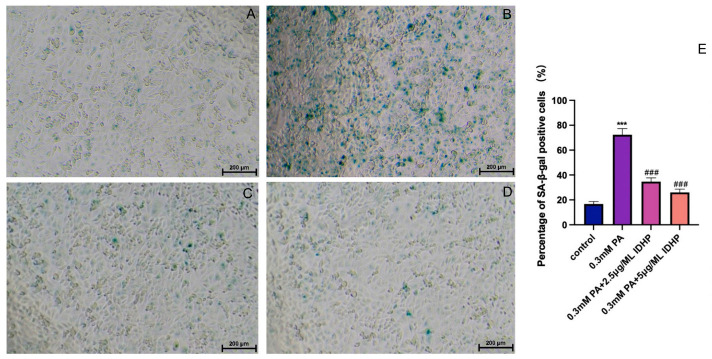
IDHP delayed PA-induced HUVEC senescence. (**A**) Control, (**B**) 0.3 mM PA, (**C**) 0.3 mM PA + 2.5 μg/ML IDHP, (**D**) 0.3 mM PA + 5 μg/ML IDHP, (**E**) The percentage of SA-β-gal positive cells was quantified by ImageJ. Data are expressed as the means ± SD. *** represents *p* < 0.001 to control group, ### represents *p* < 0.001 compared to PA group, n = 5.

First determined by MTT method is used to determine the drug concentration IDHP, as shown in Figure 2A. When IDHP concentration is below 10 µg/mL, the cell viability was higher than 90%, a value that could be considered non-cytotoxic. Therefore, IDHP below 10 µg/mL was selected for subsequent experiments. β-galactosidase staining is an important marker for assessing cellular senescence, and senescence-associated SA-β-gal positive cells show an age-dependent increase. Oxidative stress is an important factor leading to cellular senescence, and saturated fatty acid PA can induce oxidative stress. Compared with the control group, the number of SA-β-gal positive cells was significantly increased after 24 h of treatment with 0.3 mM PA. In addition, treatment with 2.5 and 5 µg/mL IDHP significantly reduced the number of positive cells in a concentration-dependent manner (Figure 3C,D).

### 2.3. Cellular ROS Experimental Results

**Figure 4:**
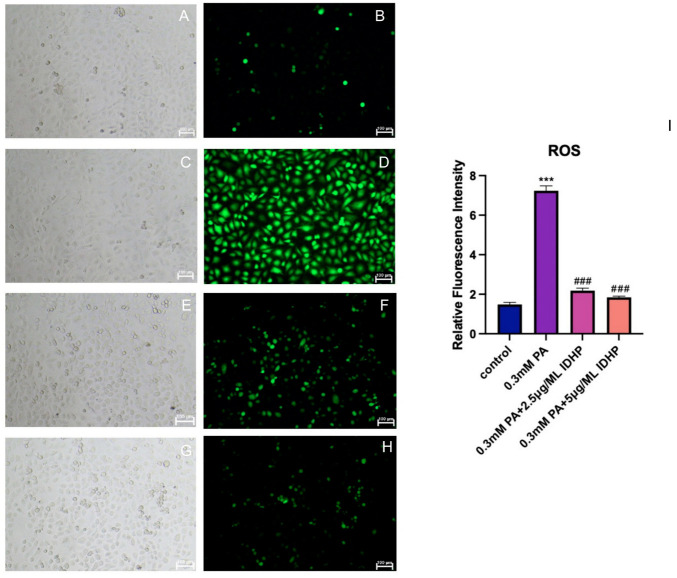
IDHP delayed PA-induced HUVEC production of ROS, and IDHP reduced ROS in a concentration-dependent manner. Under white light field of view: (**A**) Control, (**C**) 0.3 mM PA, (**E**) 0.3 mM PA + 2.5 μg/ML IDHP, (**G**) 0.3 mM PA + 5 μg/ML IDHP; Under fluorescence: (**B**) Control, (**D**) 0.3 mM PA, (**F**) 0.3 mM PA + 2.5 μg/ML IDHP, (**H**) 0.3 mM PA + 5 μg/ML IDHP; (**I**) ROS was quantified using ImageJ. Data are expressed as the means ± SD. *** represents *p* < 0.001 to control group, ### represents *p* < 0.001 compared to PA group, n = 5.

Excess ROS will accelerate the progress of aging, so whether it can reduce excess ROS is an important indicator of whether a drug can resist aging [28,29]. As shown in Figure 4B,D, the ROS produced by PA-induced vascular senescence cells was significantly increased compared with the cells in the control group. Then, as shown in Figure 4F,H, after IDHP administration, the production of ROS was significantly reduced, and the relationship was dose-dependent. When IDHP is at 2.5 μg/ML, it can effectively reduce the amount of ROS, but when IDHP is at 5 μg/ML, the effect of reducing ROS is more significant than when IDHP is at 2.5 μg/ML. This shows that through the detection experiment of the ROS fluorescent probe DCFH-DA, we can prove that IDHP can alleviate the increase in ROS levels caused by PA, reduce the damage of intracellular ROS, and maintain the cell state. Through experiments, it was found that IDHP can delay cell aging.

### 2.4. GSH Detection

**Figure 5:**
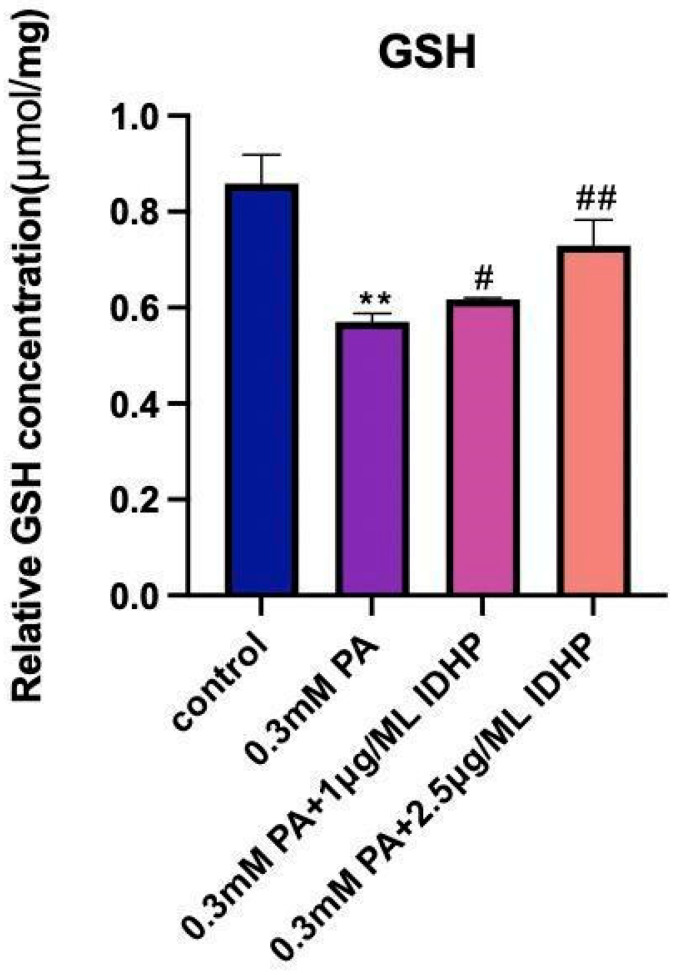
Reduced glutathione levels. ** represents *p* < 0.01 to control group, # represents *p* < 0.05 compared to PA group, ## represents *p* < 0.01 compared to PA group, n = 5.

The Glutathione (GSH) levels in HUVEC cells were detected. The results are shown in Figure 5. GSH is the most abundant antioxidant in cells and can scavenge intracellular ROS under the catalysis of glutathione peroxidase 4 (GPX4). Its content is negatively correlated with ferroptosis. The GSH levels in the PA group were significantly lower than those in the control group. After using IDHP, GSH levels increased, and the degree of increase in GSH levels was positively correlated with the concentration of IDHP. IDHP can increase GSH and alleviate cellular ferroptosis.

### 2.5. Fe^2+^ Detection

**Figure 6:**
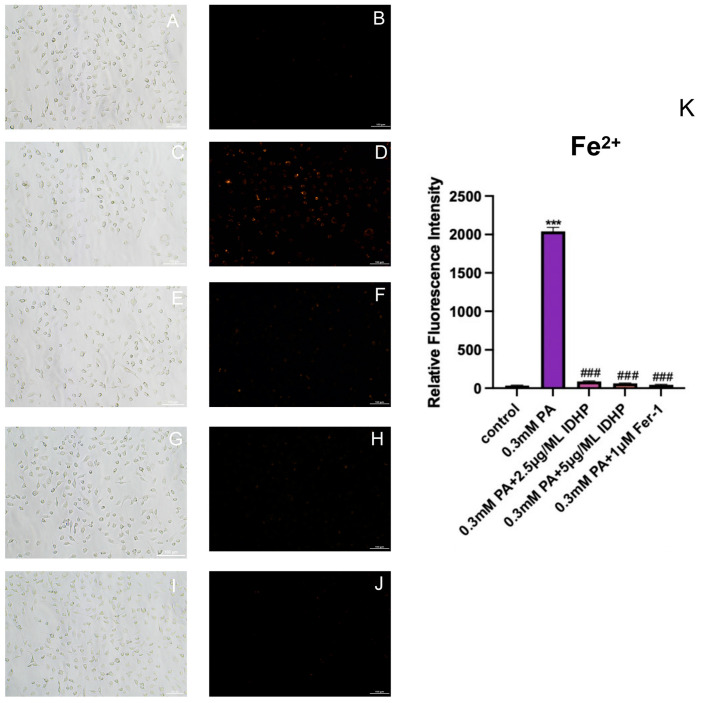
IDHP and Fer-1 can slow down the accumulation of ferrous ions. Under white light field: (**A**) Control, (**C**) 0.3 mM PA, (**E**) 0.3 mM PA + 2.5 μg/ML IDHP, (**G**) 0.3 mM PA + 5 μg/ML IDHP, (**I**) 0.3 mM PA + 1 μM Fer-1. Under fluorescence field: (**B**) Control, (**D**) 0.3 mM PA, (**F**) 0.3 mM PA + 2.5 μg/ML IDHP, (**H**) 0.3 mM PA + 5 μg/ML IDHP, (**J**) 0.3 mM PA + 1 μM Fer-1. (**K**) The degree of ferrous ion accumulation was quantitatively analyzed by ImageJ. Data are expressed as the means ± SD. *** represents *p* < 0.001 to control group, ### represents *p* < 0.001 compared to PA group, n = 5.

The intracellular ferrous ion levels in HUVEC cells under different treatments were measured using the ferrous ion fluorescent probe FeRhoNox-1, and the results are shown in Figure 6B,D; the ferrous ion levels were significantly increased after the use of PA. After processing using IDHP and Fer-1, as shown in Figure 6F,H,J, ferrous ions in cells were significantly reduced, and dose dependent IDHP is lower levels of ferrous ions. These results indicated that IDHP and Fer-1 could reduce the increase of ferrous ion level induced by PA and alleviate the ferroptosis of cells.

### 2.6. Detection of Lipid ROS

**Figure 7:**
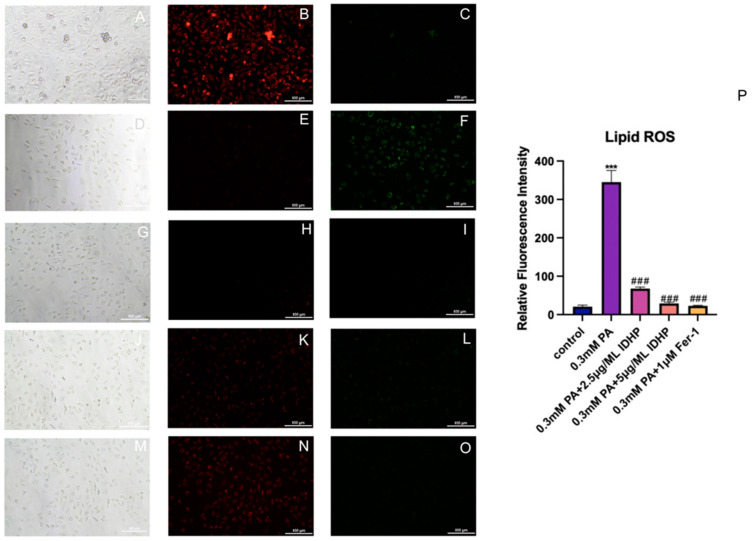
IDHP and Fer-1 can slow the extent of ferroptosis by slowing lipid peroxidation. Under the white light field: (**A**) Control, (**D**) 0.3 mM PA, (**G**) 0.3 mM PA + 2.5 μg/ML IDHP, (**J**) 0.3 mM PA + 5 mu g/ML IDHP, (**M**) 0.3 mM PA + 1 μM Fer-1. Under the red fluorescent field: (**B**) Control, (**E**) 0.3 mM PA, (**H**) 0.3 mM PA + 2.5 ug/ML IDHP, (**K**) 0.3 mM PA + 5 ug/ML IDHP, (**N**) 0.3 mM PA + 1 uM Fer-1. Under the green fluorescent field: (**C**) Control, (**F**) 0.3 mM PA, (**I**) 0.3 mM PA + 2.5 ug/ML IDHP, (**L**) 0.3 mM PA + 5 μg/ML IDHP, (**O**) 0.3 mM PA + 1 μM Fer-1. (**P**) Lipid ROS was quantified by ImageJ. Data are expressed as the means ± SD. *** represents *p* < 0.001 to control group, ### represents *p* < 0.001 compared to PA group, n = 5.

Lipid ROS fluorescence probe C11 BODIPY 581/591 was used to measure the changes of Lipid ROS in HUVEC cells under different treatments, and the results are shown in Figure 7B,C,E,F. The level of Lipid ROS in the PA group was significantly higher than that in the control group. After treatment with IDHP and Fer-1, the intracellular Lipid ROS level was significantly reduced, and IDHP reduced the Lipid ROS level in a dose-dependent manner, as shown in Figure 7H,I,K,L, indicating that IDHP and Fer-1 could reduce the increase in Lipid ROS level caused by PA. Reduce intracellular Lipid ROS damage.

### 2.7. Flow Cytometry to Detect Cell Proliferation Rate

**Figure 8:**
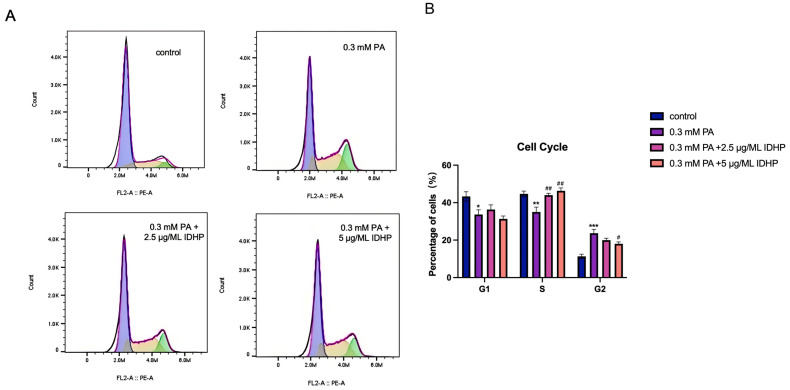
IDHP alleviates cell aging of vascular endothelial cells induced by PA. (**A**) Cell cycle flow cytometry results; (**B**) cell cycle proportion. Data are expressed as the means ± SD. * represents *p* < 0.05 compared to control group, ** represents *p* < 0.01 to control group, *** represents *p* < 0.001 to control group, # represents *p* < 0.05 compared to PA group, ## represents *p* < 0.01 compared to PA group, n = 5.

An important feature of cell senescence is to detect the proportion of cell cycle. We used flow cytometry to detect the proportion of apoptosis in different groups. We found that compared with the control group, cells administered with 0.3 mM PA experienced significant senescence and death. Then, when IDHP was used, cell cycle was significantly alleviated. Moreover, IDHP alleviated cell senescence in a dose-dependent manner, which showed that IDHP could reverse cell senescence caused by PA. As shown in Figure 8.

### 2.8. Western Blot

**Figure 9:**
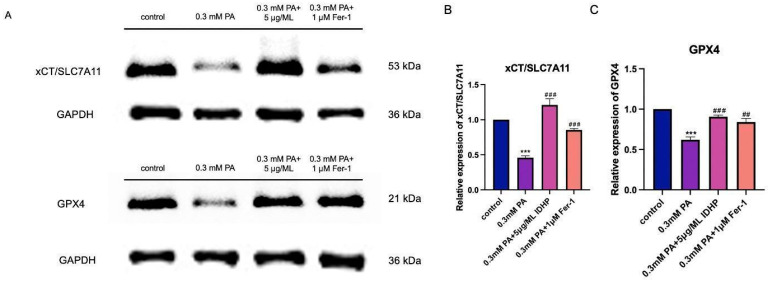
IDHP can increase the expression of GPX4 and xCT/SLC7A11 and inhibit ferroptosis. (**A**) WB original images of GPX4 and xCT/SLC7A11, (**B**) relative expression of xCT/SLC7A11, (**C**) relative expression of GPX4. Data are expressed as the means ± SD. *** represents *p* < 0.001 to control group, ### represents *p* < 0.001 compared to PA group, ## represents *p* < 0.01 compared to PA group, n = 5.

Two important proteins in the ferroptosis pathway are GPX4 and xCT/SLC7A11, which are involved in ferroptosis. xCT/SLC7A11 usually protects cells from cell death induced by various cellular stresses and can inhibit ferroptosis. xCT/SLC7A11 promotes the production of reduced GSH. GPX4, as a ROS detoxification enzyme, can convert accumulated lipid hydroperoxides in cells into lipid alcohols, reduce peroxide-related products, and thereby protect cells from damage caused by ROS. Therefore, GPX4 plays a crucial role in inhibiting ferroptosis. We used Western Blot to detect the expression of GPX4 and xCT/SLC7A11. We found that PA can reduce the expression of GPX4 and xCT/SLC7A11, the key proteins of ferroptosis. Furthermore, IDHP can increase the expression of GPX4 and xCT/SLC7A11. The experimental results indicate that IDHP can alleviate ferroptosis caused by PA. As shown in Figure 9.

## 3. Discussion

Vascular aging is one of the important causes of cardiovascular disease [30]. Vascular aging exacerbates changes in vascular structure and function, thereby increasing the risk of cardiovascular disease [31]. In previous studies, our group found that certain drugs reduce the lipotoxicity of cardiomyocytes through mTOR/HIF-1α dilution and can also resist cardiomyopathy through ROS/p38/JNK coupling [32].

As a type of palmitic acid, PA can cause an increase in fat in vascular cells and aggravate ferroptosis, which has been confirmed in previous papers [33]. IDHP has a wide range of treatments, and a large number of studies have shown that IDHP has great therapeutic potential for cardiovascular diseases [25,26]. PA affects intracellular iron content by inducing endoplasmic reticulum (ER) stress. This excess iron can lead to ferroptosis [34]. PA will stimulate the increase of intracellular ROS production, and the degree of intracellular lipid peroxidation will also be aggravated, resulting in an imbalance of intracellular redox homeostasis and leading to intracellular ferroptosis [35]. IDHP reduces the production of mitochondrial ROS, balances the intracellular redox imbalance caused by PA induction, inhibits the accumulation of ferrous ions, and reduces the degree of lipid peroxidation to slow down ferroptosis.

Besides, IDHP has antioxidant activity. It is speculated that IDHP may reduce vascular aging caused by fat accumulation and play a protective role [26]. Previous studies have found that IDHP can delay cellular aging by regulating the GAS6/Axl-AMPK signaling pathway [27]. These studies have revealed to us the potential role of IDHP in delaying aging. However, the specific mechanism of this effect is not particularly clear. Therefore, in this study, we elaborated on the role of IDHP in resisting vascular aging.

Age-related increases in the levels of endogenous oxidative stress activate the ferroptosis pathway to further accelerate the aging process and the appearance of atherosclerosis-related pathological features in aging tissues [36]. In our study, we investigated whether IDHP could delay vascular aging and reduce the incidence of CVD. IDHP has been found to reduce the excessive ROS, thus preliminarily confirming that IDHP retards vascular cell senescence. To further investigate the effect of IDHP on vascular aging, we investigated the effect of IDHP on ferroptosis in cells. We demonstrate that IDHP significantly reduces Fe^2+^ and Lipid ROS, and IDHP can also reduce GSH. In addition, IDHP also affects the cell cycle. These results suggest that IDHP is able to delay cellular senescence by inhibiting ferroptosis.

Fer-1 as a death inhibitor, works through the following mechanisms: inhibition of lipid peroxidation. Fer-1 can inhibit lipid peroxidation, reduce the accumulation of lipid peroxide. It captures free radicals and stabilizes lipid peroxide, to prevent further oxidation reactions, thus protecting cells against cell damage caused by lipid peroxide; Adjust the metabolism of iron: Fer-1 can regulate iron metabolism in the cell. It can inhibit the accumulation and release of iron ions, prevent excessive free iron ions from participating in oxidation reactions, and thus reduce the occurrence of oxidative stress [37]. In our experiments, we also used ferroptosis inhibitors. In future experiments, we can also choose other ferroptosis inhibitors to increase the depth of research on the pathways through which IDHP can resist aging.

Our study has implications for IDHP’s ability to reverse the ROS/ferroptosis pathway and reveals the potential of IDHP as a compound to resist vascular aging and CVD. We think that future research can conduct more research on other mechanisms of IDHP in resisting vascular aging and cardiovascular diseases.

## 4. Materials and Methods

### 4.1. Cell Line and Culture

HUVEC cells were purchased from the Cell Resource Center, Shanghai Institute of Life Sciences, Chinese Academy of Sciences. Cells were placed in a carbon dioxide incubator (37 °C, 5% CO_2_) for culture. The cell medium consisted of 90% DMEM (Thermo Fisher Scientific/Gibco, Waltham, MA, USA) + 9% FBS fetal bovine serum + 1% double antibody (Penicillin-streptomycin) (Thermo Fisher Scientific/Gibco, Waltham, MA, USA). PA was prepared by using sterilized 5% bovine serum albumin (BSA, Biyuntian, Shanghai, China; g/mL, BSA/PBS) solution was gradually diluted to obtain 62.5 mM PA stock solution, which was stored at −20 °C for further determination. IDHP was from Northwestern University and Fer-1 was purchased from MCE Corporation. Cells were seeded for 12 h. Then PA, IDHP and ferroptosis inhibitor Fer-1 were administered for 24 h. The final concentration of DMSO in each group was 0.1% (*v*/*v*) and the mass fraction of BSA was 5% (g/mL). PA and drugs were used to establish the model and drug treatment, and then they were cultured in a carbon dioxide incubator for 24 h. The corresponding samples were then collected for further experiments.

### 4.2. Cell Viability—MTT Assay

Cell viability was assayed using MTT (3-(4,5-dimethylthiazol-2-yl)-2,5-diphenyltetrazolium bromide) (Coolaber, Beijing, China). MTT was prepared in sterile PBS as a 5 mg/mL stock solution, fully dissolved and then filtered for sterilization using a 0.22 μm needle filter (PALL, New York, NY, USA). Cells were inoculated in 96-well plates. After 24 h of the treatment, 20 μL of the MTT solution was added to each well and further incubated at 37 °C for 4 h in the cell culture incubator. Then, the cell medium was removed, the formed formazan was dissolved with 200 μL DMSO solution, and the absorbance at 490 nm was analyzed with an Epoch microplate spectrophotometer (Bio-Tek, Winooski, VT, USA).

### 4.3. Senescence-Associated β-Galactosidase Staining Assay

The SA β-galactosidase staining kit (Biyuntian, Shanghai, China) was used to detect the activity of senescence-specific β-galactosidase. The cells were inoculated in 96-well plates for 24 h. The cell culture was aspirated, washed once with PBS, treated with 100 μL of β-galactosidase staining fixative and fixed for 15 min at room temperature. The 1000 μL cell-staining working solution consisted of 10 μL β-galactosidase staining solution A, 10 μL β-galactosidase staining solution B, 930 μL β-galactosidase staining solution C and 50 μL X-Gal solution. After the cell fixative was aspirated, the cells were washed with PBS thrice for 3 min each time. Then, we aspirated the PBS and added 100 μL of staining working solution to each well. We sealed the 96-well plates using a sealing film and incubated them overnight at 37 °C in a CO_2_-free incubator for observation under a Leica DMI6000 B inverted microscope (Leica Microsystems, Wetzlar, Germany). Cell counts were performed using ImageJ software (National Institutes of Health, Bethesda, MD, USA), and quantitative results were obtained by dividing the number of positive cells in the field of view by the total number of cells.

### 4.4. Cellular ROS Assay—DCFH-DA Probe

Cells were inoculated in 96-well plates for 24 h. A DCFH-DA probe (Biyuntian, Shanghai, China) was diluted with serum-free culture medium at 1:1000 to a final concentration of 10 μmol/L. The cell culture medium was removed, the diluted probe was added and the cells were incubated at 37 °C for 20 min in a cell incubator. The medium was aspirated and washed three times with PBS, and the cells were observed and photographed under a fluorescent microscope (Leica Microsystems, Wetzlar, Germany).

For ROS quantification experiments, cells were seeded in 24-well plates and subjected to the same treatment as described above. In fluorescent inverted microscope (Leica Microsystems, Wetzlar, Germany) under observation. Then use the ImageJ for quantitative analysis.

### 4.5. Lipid ROS Detection

C11 BODIPY 581/591 lipid peroxidation fluorescent probe (Mao-Kang, Shanghai, China) was used for the experiments. The cells were seeded at 1 × 10^4^ cells/well with 200 μL of cell suspension and cultured in 96-well plates for 24 h. After completion of the culture, DMEM medium was removed by aspiration and washed three times with PBS, then 100 μL of diluted fluorescent probe (1:1000 PBS) was added to each well and incubated for 1 h in a CO_2_ cell incubator. The culture medium was removed by aspiration, and 200 μL PBS was added to each well after washing three times with PBS. The cells were photographed by fluorescence microscopy. In this experiment, try to avoid light as much as possible to slow down fluorescence quenching.

### 4.6. Fe^2+^ Detection

FeRhoNox-1 ferroptosis fluorescent probe (Mao-Kang, Shanghai, China) was used for the experiments. FeRhoNox-1, also known as RhoNox-1, is an active fluorescent probe that specifically detects labile iron (II) ions (Fe^2+^). Once reacted with Fe^2+^, an orange (red) fluorescent product is irreversibly generated (Absmax = 540 nm, FLmax = 575 nm, spectral characteristics of FeRhoNox-1). Iron (III) ions (Fe^3+^) or other divalent metal ions other than iron ions at physiological concentrations will not enhance its fluorescence. FeRhoNox-1 has cell membrane permeability and high selectivity. It is suitable for the detection of Fe^2+^ in living cells and tends to be localized in the Golgi apparatus. 200 μL cell suspension was seeded in 96-well plates at 1 × 10^4^ cells/well. After incubation, DMEM medium was removed by suction, washed three times with PBS, and then 100 μL of probe working solution (FeRhoNox-1 ferrous ion fluorescent probe stock solution was diluted in PBS by 1000-fold) was added to each well. After incubating for 1 h in a carbon dioxide cell incubator, photographs were taken by fluorescence microscopy and analyzed. In this experiment, try to avoid light as much as possible to slow down fluorescence quenching.

### 4.7. GSH Detection

The study used GSH and GSSG detection kit (Biyuntian, Shanghai, China). Cells were seeded in 6-well plates at 1.5 × 10^5^ cells/well. After culture, DMEM medium was removed by suction, washed once with PBS, 500 μL trypsin was added to each well for digestion for 30 s, cells were blown off by pipetting gun, cells were resuspended and washed twice with PBS after centrifugation. Then, according to the wet weight of the cells (10 mg cells can be regarded as 10 μL), the reagent of protein scavenger M in the triploid volume was added according to the instructions of the kit, and the samples were quickly frozen and thawed twice using liquid nitrogen and 37 °C water bath. The samples were then centrifuged at 12,000 rpm for 10 min at 4 °C to remove the supernatant for determination. Then we took some of the samples prepared above to be tested for total glutathione content, added diluted GSH scavenging auxiliary solution at a ratio of 20 μL of diluted GSH scavenging auxiliary solution per 100 μL of sample, and mixed immediately with Vortex. Then add GSH clearing reagent working solution at a ratio of 4 μL GSH clearing reagent working solution per 100 μL sample, vortex and mix immediately, and react at 25 °C for 60 min. And then the absorbance at 412 nm was measured every 5 min by microplate reader. Finally, the absorbance was compared with the standard curve to calculate the GSH content.

### 4.8. Flow Cytometry

For the flow cytometry assay, cells were seeded into 6-well plates. After treatment, the cell medium was collected, and then the cells were digested and centrifuged at 800 rpm for 3 min with the cell medium, incubating with Calcein-AM/PI Double Stain Kit (Yisheng, Shanghai, China) reagent for 20 min. Then flow cytometry was used for cell cycle determination, and finally ImageJ were used for data analysis and processing.

### 4.9. Western Blot

The cells were inoculated in 6-well plates for 24 h. Then, the culture dishes were placed on ice and washed twice with ice-cold PBS. Cell lysate (Biyuntian, Shanghai, China) reagent was added and cell lysis was collected into EP tubes. Then, cell lysis was centrifuged at 12,000 × *g* for 10 min at 4 °C and the supernatant was collected. Sample preparation was performed after determination of the supernatant protein concentration with the Bradford assay (Biyuntian, Shanghai, China). Subsequently, equal amounts of protein (30 μg/lane) were separated using 12.5% sodium dodecyl sulfate polyacrylamide electrophoresis gel (Epizyme Biotech, Shanghai, China) and then transferred to nitrocellulose membranes (Pall, New York, NY, USA). The nitrocellulose membranes with the transferred proteins were then incubated for 2 h at room temperature using 5% skim milk (Epizyme Biomedical Technology Co., Ltd., Shanghai, China), incubated overnight at 4 °C. And we used primary antibodies and visualized using enhanced luminescence solution (Thermo Fisher Scientific Inc., Waltham, MA, USA) the following day after 2 h incubation using secondary antibodies. The specific primary antibodies used for the Western blot analysis against protein—namely, GAPDH(1:1000), GPX4(1:1000), xCT/SLC7A11(1:1000). GAPDH were purchased from ABSIN company ( Shanghai, China). Besides, GPX4 and xCT/SLC7A11 were purchased from Cell Signal Technology (Danvers, MA, USA). At the end of incubation with the primary antibody, the membranes were washed three times for 10 min each by shaking on a shaker using 1 × TBST. After washing, the membranes were placed in the secondary antibody solutions corresponding to the primary antibodies (Goat Anti-Mouse IgG or Goat Anti-Rabbit IgG, both purchased from Cell Signal Technology) and incubated for 2 h. At the end of incubation with secondary antibodies, the membranes were washed three times for 20 min each with 1 × TBST while shaking on a shaker and visualized using a luminescent solution (Thermo Fisher Scientific Inc., Waltham, MA, USA), and then the gray density of protein bands was analyzed using ImageJ software.

### 4.10. Statistical Analysis

GraphPad Prism 9.0 software (GraphPad Software, San Diego, CA, USA) was used for the statistical analysis. All the experiments were repeated three or more times independently and the data are expressed as means ± standard deviation (SD). Differences between groups with statistical significance were calculated using ANOVA followed by Tukey’s post hoc test. *p* < 0.05 was considered statistically significant.

## 5. Conclusions

**Figure 10:**
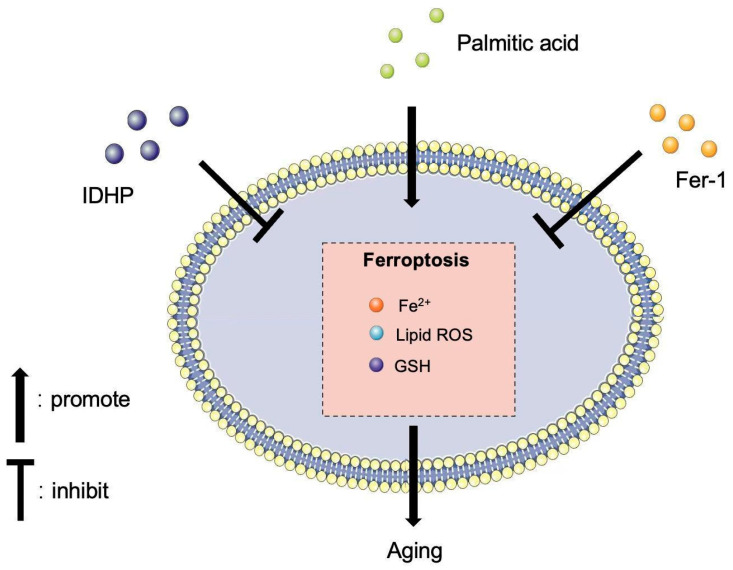
IDHP inhibits PA-induced replicative cellular senescence via the ROS/ferroptosis pathway.

In conclusion, our study for the first time revealed the mechanism of action of IDHP in delaying aging through an in vitro model, thus providing a theoretical basis for the application of IDHP in delaying aging. Our work determined the IDHP, ROS, and the mechanism of ferroptosis, and provides the theoretical basis for the use of ROS/ferroptosis pathways in regulating cell life, as shown in Figure 10. This study gives IDHP new medicinal value and offers a new treatment for vascular senescence.

## Data Availability

The data presented in this study are available. If you need detailed data, you need to contact the corresponding author.

## References

[1] Chen J., Ma D., He L., Yang D. (2024). Senolystics from natural products for extending health and lifespan. Tradit. Med. Res..

[2] Pearson K.J., Baur J.A., Lewis K.N., Peshkin L., Price N.L., Labinskyy N., Swindell W.R., Kamara D., Minor R.K., Perez E. (2008). Resveratrol delays age-related deterioration and mimics transcriptional aspects of dietary restriction without extending life span. Cell Metab..

[3] Huber T.S., Björck M., Chandra A., Clouse W.D., Dalsing M.C., Oderich G.S., Smeds M.R., Murad M.H. (2021). Chronic mesenteric ischemia: Clinical practice guidelines from the Society for Vascular Surgery. J. Vasc. Surg..

[4] Lin T., Yang W.Q., Luo W.W., Zhang L.L., Mai Y.Q., Li Z.Q., Liu S.T., Jiang L.J., Liu P.Q., Li Z.M. (2022). Disturbance of Fatty Acid Metabolism Promoted Vascular Endothelial Cell Senescence via Acetyl-CoA-Induced Protein Acetylation Modification. Oxid. Med. Cell Longev..

[5] Hwang H.J., Kim N., Herman A.B., Gorospe M., Lee J.S. (2022). Factors and Pathways Modulating Endothelial Cell Senescence in Vascular Aging. Int. J. Mol. Sci..

[6] Lakatta E.G., Levy D. (2003). Arterial and cardiac aging: Major shareholders in cardiovascular disease enterprises: Part I: Aging arteries: A “set up” for vascular disease. Circulation.

[7] Robert L., Molinari J., Ravelojaona V., Andrès E., Robert A.M. (2010). Age- and passage-dependent upregulation of fibroblast elastase-type endopeptidase activity. Role of advanced glycation endproducts, inhibition by fucose- and rhamnose-rich oligosaccharides. Arch. Gerontol. Geriatr..

[8] Kuilman T., Michaloglou C., Mooi W.J., Peeper D.S. (2010). The essence of senescence. Genes. Dev..

[9] Wang Y., Wang J., Zheng X.W., Du M.F., Zhang X., Chu C., Wang D., Liao Y.Y., Ma Q., Jia H. (2023). Early-Life Cardiovascular Risk Factor Trajectories and Vascular Aging in Midlife: A 30-Year Prospective Cohort Study. Hypertension.

[10] Ji H., Kwan A.C., Chen M.T., Ouyang D., Ebinger J.E., Bell S.P., Niiranen T.J., Bello N.A., Cheng S. (2022). Sex Differences in Myocardial and Vascular Aging. Circ. Res..

[11] Jankowski J., Floege J., Fliser D., Böhm M., Marx N. (2021). Cardiovascular Disease in Chronic Kidney Disease: Pathophysiological Insights and Therapeutic Options. Circulation.

[12] Louka A.M., Sagris D., Ntaios G. (2022). Immunity, Vascular Aging and Stroke. Curr. Med. Chem..

[13] Tyrrell D.J., Blin M.G., Song J., Wood S.C., Zhang M., Beard D.A., Goldstein D.R. (2020). Age-Associated Mitochondrial Dysfunction Accelerates Atherogenesis. Circ. Res..

[14] Dixon S.J., Olzmann J.A. (2024). The cell biology of ferroptosis. Nat. Rev. Mol. Cell Biol..

[15] Newton K., Strasser A., Kayagaki N., Dixit V.M. (2024). Cell death. Cell.

[16] Li C., Liu R., Xiong Z., Bao X., Liang S., Zeng H., Jin W., Gong Q., Liu L., Guo J. (2024). Ferroptosis: A potential target for the treatment of atherosclerosis. Acta Biochim. Biophys. Sin..

[17] Liu C., Shen Y., Cavdar O., Huang J., Fang H. (2023). Angiotensin II-induced vascular endothelial cells ferroptosis via P53-ALOX12 signal axis. Clin. Exp. Hypertens..

[18] Li Y., Zhang E., Yang H., Chen Y., Tao L., Xu Y., Chen T., Shen X. (2022). Gastrodin Ameliorates Cognitive Dysfunction in Vascular Dementia Rats by Suppressing Ferroptosis via the Regulation of the Nrf2/Keap1-GPx4 Signaling Pathway. Molecules.

[19] Shao S., Liu Y., Hong W., Mo Y., Shu F., Jiang L., Tan N. (2023). Influence of FOSL1 Inhibition on Vascular Calcification and ROS Generation through Ferroptosis via P53-SLC7A11 Axis. Biomedicines.

[20] Zheng D., Liu J., Piao H., Zhu Z., Wei R., Liu K. (2022). ROS-triggered endothelial cell death mechanisms: Focus on pyroptosis, parthanatos, and ferroptosis. Front. Immunol..

[21] Battaglia A.M., Chirillo R., Aversa I., Sacco A., Costanzo F., Biamonte F. (2020). Ferroptosis and Cancer: Mitochondria Meet the “Iron Maiden” Cell Death. Cells.

[22] Wu S., Mao C., Kondiparthi L., Poyurovsky M.V., Olszewski K., Gan B. (2022). A ferroptosis defense mechanism mediated by glycerol-3-phosphate dehydrogenase 2 in mitochondria. Proc. Natl. Acad. Sci. USA.

[23] Feng F., He S., Li X., He J., Luo L. (2024). Mitochondria-mediated Ferroptosis in Diseases Therapy: From Molecular Mechanisms to Implications. Aging Dis..

[24] Wang X., Zhao L., Wang C., Wang L., Wu H., Song X., Wang W., Xu H., Dong X. (2023). Potent nanoreactor-mediated ferroptosis-based strategy for the reversal of cancer chemoresistance to Sorafenib. Acta Biomater..

[25] Zhang M.L., Wang M., Chen J., Liu Y.J., Yu Y.J., Liu L.M., Zheng X.H., Xiao Y.C., Zhang J.M., Zhu M.X. (2023). Isopropyl 3-(3, 4-dihydroxyphenyl)-2-hydroxypropanoate protects lipopolysaccharide-induced acute lung injury in mice by attenuating pyroptosis. Eur. J. Pharmacol..

[26] Wang S., Wang H., Jing H., Wang S., Kang L., Gao X., Hu L., Zheng X. (2012). Anti-inflammatory effects of isopropyl 3-(3, 4-dihydroxyphenyl)-2-hydroxypropanoate, a novel metabolite from danshen, on activated microglia. Chin. J. Physiol..

[27] Lei W., Xu X., Li N., Zhang Y., Tang R., Li X., Tang J., Wu X., Lu C., Bai Y. (2024). Isopropyl 3-(3,4-dihydroxyphenyl) 2-hydroxypropanoate protects septic myocardial injury via regulating GAS6/Axl-AMPK signaling pathway. Biochem. Pharmacol..

[28] Baranov V.S., Baranova E.V. (2017). Aging and Ambiguous ROS. System Genetics Analysis. Curr. Aging Sci..

[29] Ungvari Z., Tarantini S., Donato A.J., Galvan V., Csiszar A. (2018). Mechanisms of Vascular Aging. Circ. Res..

[30] Borlaug B.A., Sharma K., Shah S.J., Ho J.E. (2023). Heart Failure With Preserved Ejection Fraction: JACC Scientific Statement. J. Am. Coll. Cardiol..

[31] Faleeva M., Ahmad S., Theofilatos K., Lynham S., Watson G., Whitehead M., Marhuenda E., Iskratsch T., Cox S., Shanahan C.M. (2024). Sox9 Accelerates Vascular Aging by Regulating Extracellular Matrix Composition and Stiffness. Circ. Res..

[32] Hao W., Shan W., Wan F., Luo J., Niu Y., Zhou J., Zhang Y., Xu N., Xie W. (2023). Canagliflozin Delays Aging of HUVECs Induced by Palmitic Acid via the ROS/p38/JNK Pathway. Antioxidants.

[33] Wan F., He X., Xie W. (2024). Canagliflozin Inhibits Palmitic Acid-Induced Vascular Cell Aging In Vitro through ROS/ERK and Ferroptosis Pathways. Antioxidants.

[34] Kuang H., Sun X., Liu Y., Tang M., Wei Y., Shi Y., Li R., Xiao G., Kang J., Wang F. (2023). Palmitic acid-induced ferroptosis via CD36 activates ER stress to break calcium-iron balance in colon cancer cells. FEBS J..

[35] Tan X.H., Gu Y.Y., Song W.P., Nan T.G., Song W.D., Fang D., Yuan Y.M., Xin Z.C., Li X.S., Guan R.L. (2023). Transcriptome analysis highlights the role of ferroptosis in palmitic acid-induced endothelial dysfunction. Sex. Med..

[36] Tong Y., Wu Y., Ma J., Ikeda M., Ide T., Griffin C.T., Ding X.Q., Wang S. (2023). Comparative mechanistic study of RPE cell death induced by different oxidative stresses. Redox Biol..

[37] Wang K., Jiang L., Zhong Y., Zhang Y., Yin Q., Li S., Zhang X., Han H., Yao K. (2022). Ferrostatin-1-loaded liposome for treatment of corneal alkali burn via targeting ferroptosis. Bioeng. Transl. Med..

